# Functional Interpretation of a Non-Gut Hemocoelic Tissue Aminopeptidase N (APN) in a Lepidopteran Insect Pest *Achaea janata*

**DOI:** 10.1371/journal.pone.0079468

**Published:** 2013-11-14

**Authors:** Thuirei Jacob Ningshen, Polamarasetty Aparoy, Venkat Rao Ventaku, Aparna Dutta-Gupta

**Affiliations:** 1 Department of Animal Sciences, School of Life Sciences, University of Hyderabad, Hyderabad, Andhra Pradesh, India; 2 Centre for Computational Biology and Bioinformatics, School of Life Sciences, Central University of Himachal Pradesh, Dharamshala, Himachal Pradesh, India; The Ohio State University/OARDC, United States of America

## Abstract

Insect midgut membrane-anchored aminopeptidases N **(**APNs) are Zn^++^ dependent metalloproteases. Their primary role in dietary protein digestion and also as receptors in Cry toxin-induced pathogenesis is well documented. APN expression in few non-gut hemocoelic tissues of lepidopteran insects has also been reported but their functions are widely unknown. In the present study, we observed specific *in vitro* interaction of Cry1Aa toxin with a 113 kDa AjAPN1 membrane protein of larval fat body, Malpighian tubule and salivary gland of *Achaea janata*. Analyses of 3D molecular structure of AjAPN1, the predominantly expressed APN isoform in these non-gut hemocoelic tissues of *A. janata* showed high structural similarity to the Cry1Aa toxin binding midgut APN of *Bombyx mori*, especially in the toxin binding region. Structural similarity was further substantiated by *in vitro* binding of Cry1Aa toxin. RNA interference (RNAi) resulted in significant down-regulation of *AjAPN1* transcript and protein expression in fat body and Malpighian tubule but not in salivary gland. Consequently, reduced AjAPN1 expression resulted in larval mortality, larval growth arrest, development of lethal larval-pupal intermediates, development of smaller pupae and emergence of viable defective adults. *In vitro* Cry1Aa toxin binding analysis of non-gut hemocoelic tissues of AjAPN1 knockdown larvae showed reduced interaction of Cry1Aa toxin with the 113 kDa AjAPN1 protein, correlating well with the significant silencing of AjAPN1 expression. Thus, our observations suggest AjAPN1 expression in non-gut hemocoelic tissues to play important physiological role(s) during post-embryonic development of *A. janata*. Though specific interaction of Cry1Aa toxin with AjAPN1 of non-gut hemocoelic tissues of *A. janata* was demonstrated, evidences to prove its functional role as a Cry1Aa toxin receptor will require more in-depth investigation.

## Introduction

Insect midgut aminopeptidases N (APNs) are Zn^++^ dependent gluzincin family M1 metalloproteases [1] attached to brush border membrane of the epithelial cells through a glycosylphosphatidyl-inositol (GPI) anchor [2], [3]. In midgut of lepidopteran insect larvae, APNs are primarily involved in dietary protein digestion whereby they cleave a single amino acid residue from the N-terminus of oligopeptides, preferentially the neutral amino acids [4], [5]. However, they are mainly studied for their role as receptors in Cry toxin-induced pathogenesis in insects [6], [7]. The Cry proteins produced by a gram positive bacterium *Bacillus thuringiensis* are in the form of protoxins which upon ingestion by larvae of susceptible insects, are cleaved by the midgut proteinases to form active toxins. The activated toxins then bind to specific midgut receptors resulting in oligomerization and insertion of toxins into the membranes to generate pores leading to cell lysis and finally, the death of the insect [5], [8]. Though cadherin-like proteins [9], GPI-anchored alkaline phosphatases (ALPs) [10], glycolipids [11] and glyconjugates [5] are reported receptors for Cry toxins, the GPI-anchored APNs [12], [13] by far are the most widely studied and well characterized Cry toxin receptors. Apart from midgut, APN expression in fat body [14], [15], Malpighian tubule [4], [16], [17], [18], salivary gland [18] of lepidopteran insects has now been reported. Pore forming ability of Cry toxins on *in vitro* cultured fat body cells indicated the possibility of Cry toxins binding to fat body membrane proteins and causing toxic effects to the cells [19]. Transgenic expression of *Manduca sexta* midgut APN in *Drosophila melanogaster* induced sensitivity to the lepidopteran-specific insecticidal Cry1Ac which otherwise is not toxic [20]. Further, Sivakumar *et al* also demonstrated that Sf21 insect cells expressing *Helicoverpa armigera* midgut APN which allowed high sensitivity to Cry1Ac, upon down-regulation by RNA interference (RNAi) resulted in reduced sensitivity [21]. These studies suggest the possibility of Cry toxins causing insecticidal effects on cells where APNs are expressed.

In cases where the experimental determination of protein three-dimensional (3D) structure is not possible, homology modeling is the most widely used approach. To date, there are no reports on crystal structure of insect APNs. However, molecular models of midgut-specific APNs from *M. sexta* [22] and *Spodoptera litura* [23] have been generated using homology modeling strategy. RNAi-mediated knockdown of gene expression in lepidopteran insects either by feeding or intra-hemocoelic injection has been commonly used to identify potential target genes for pest control [13], [21], [24], [25]. Gene silencing studies revealed the functional role of midgut APNs as a Cry toxin receptor in *S. litura* [13] and *H. armigera* [21]. In *A. janata*, AjAPN1 is the APN isoform which is predominantly expressed in fat body, Malpighian tubule and salivary gland [18]. However till date, proper functional characterization of non-gut hemocoelic tissue APNs in insects has been lacking.

In the present study, we employed homology modeling and RNAi strategies to decipher the functional role of AjAPN1 expression in non-gut hemocoelic tissues of *A. janata* larvae. We demonstrated specific *in vitro* interaction of Cry1Aa toxin with the 113 kDa AjAPN1 membrane protein of larval fat body, Malpighian tubule and salivary gland. High similarity of 3D molecular structure of AjAPN1 of *A. janata* with that of *Bombyx mori* midgut APN (Genbank AAC33301), especially in the Cry1Aa toxin binding region as well as *in vitro* binding of Cry1Aa toxin to it further supported its potential role in Cry toxin interaction and toxicity. RNAi-mediated silencing not only down-regulated AjAPN1 expression in fat body and Malpighian tubule but also induced adverse physiological effects, which suggest that it plays important physiological role during growth, development as well as metamorphosis in *A. janata*. *In vitro* Cry1Aa toxin binding analysis of non-gut hemocoelic tissues of AjAPN1 knockdown larvae showed drastically reduced interaction of Cry1Aa toxin with the 113 kDa AjAPN1 protein, correlating well with the significantly reduced levels of *AjAPN1* transcript and its encoded protein expression. Findings from the present study suggest AjAPN1 expression in non-gut hemocoelic tissues to play important physiological role(s) during post-embryonic development and metamorphosis of *A. janata*. Though specific interaction of Cry1Aa toxin with the 113 kDa AjAPN1 protein of non-gut hemocoelic tissues of *A. janata* was demonstrated, evidences to prove its functional role as a Cry1Aa toxin receptor in *A. janata* will require further investigation.

## Results

### *In vitro* Cry1Aa Toxin Binding Analysis

**Figure 1:**
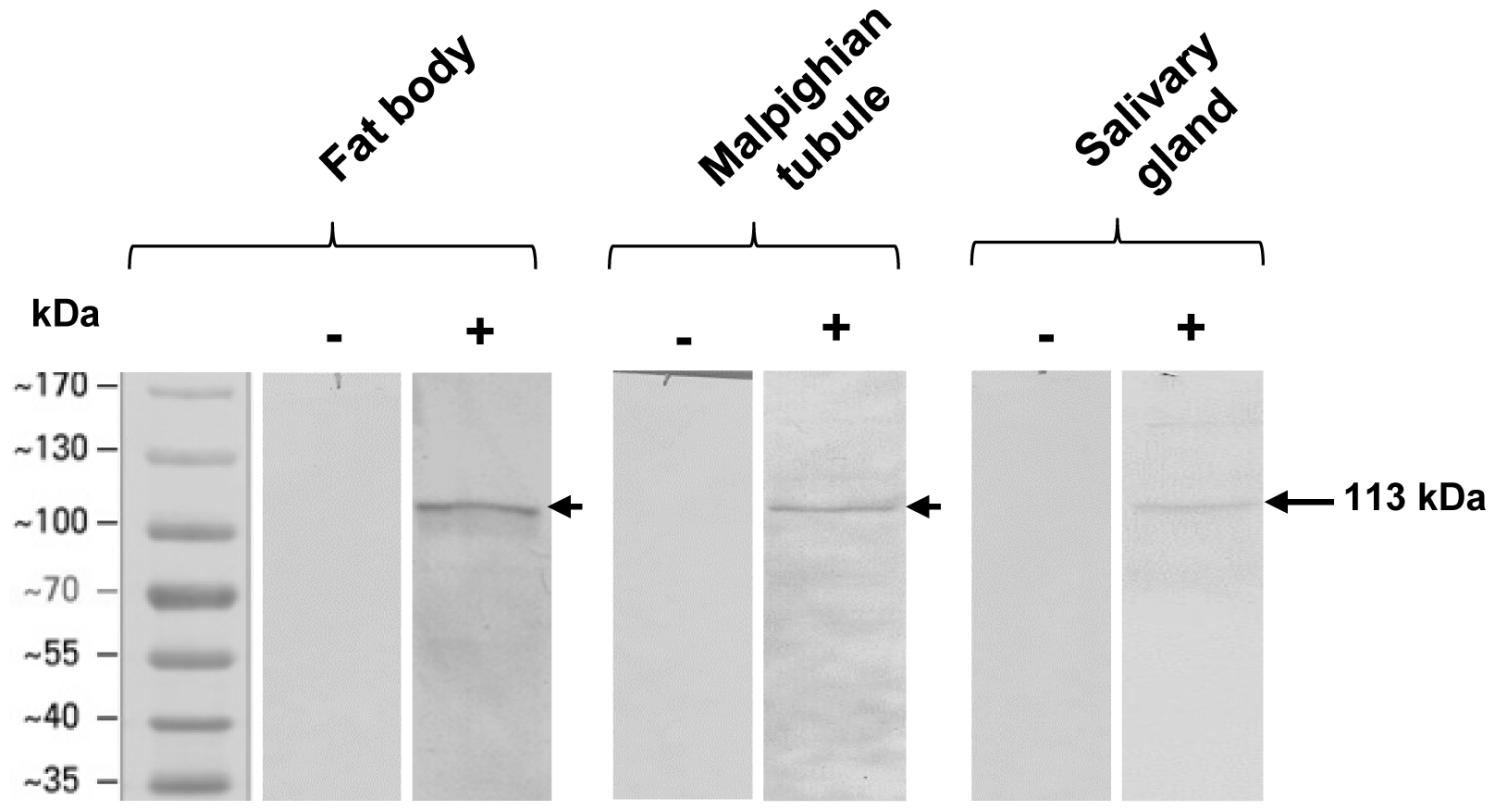
*In vitro* analysis of Cry1Aa toxin binding. 30 µg each of fat body, Malpighian tubule and salivary gland membrane protein fractions prepared from early fifth instar (5E) larvae were separated by 7.5% SDS-PAGE, transferred onto nitrocellulose membranes, then incubated with biotinylated Cry1Aa toxin (200 ng/ml), followed by incubation with streptavidin-ALP conjugate and finally developed with NBT-BCIP substrate. Note the detection of a 113 kDa interacting membrane protein in all the three tissues. Blots labeled (**−**) are the control blots incubated with unlabeled Cry1Aa toxin while those labeled (+) are the blots incubated with biotinylated Cry1Aa toxin.

The observations from ligand binding studies presented in figure 1 clearly showed strong binding of Cry1Aa toxin predominantly to a 113 kDa membrane protein of fat body, Malpighian tubule and salivary gland. However, the interaction was found to be less in salivary gland compared to the other two tissues. Control blots which were incubated with unlabeled Cry1Aa toxin did not show any signal. We also noticed weak binding of Cry1Aa toxin to few other proteins in the blots.

### Immunoprecipitation of Cry1Aa Toxin Interacting Protein

**Figure 2:**
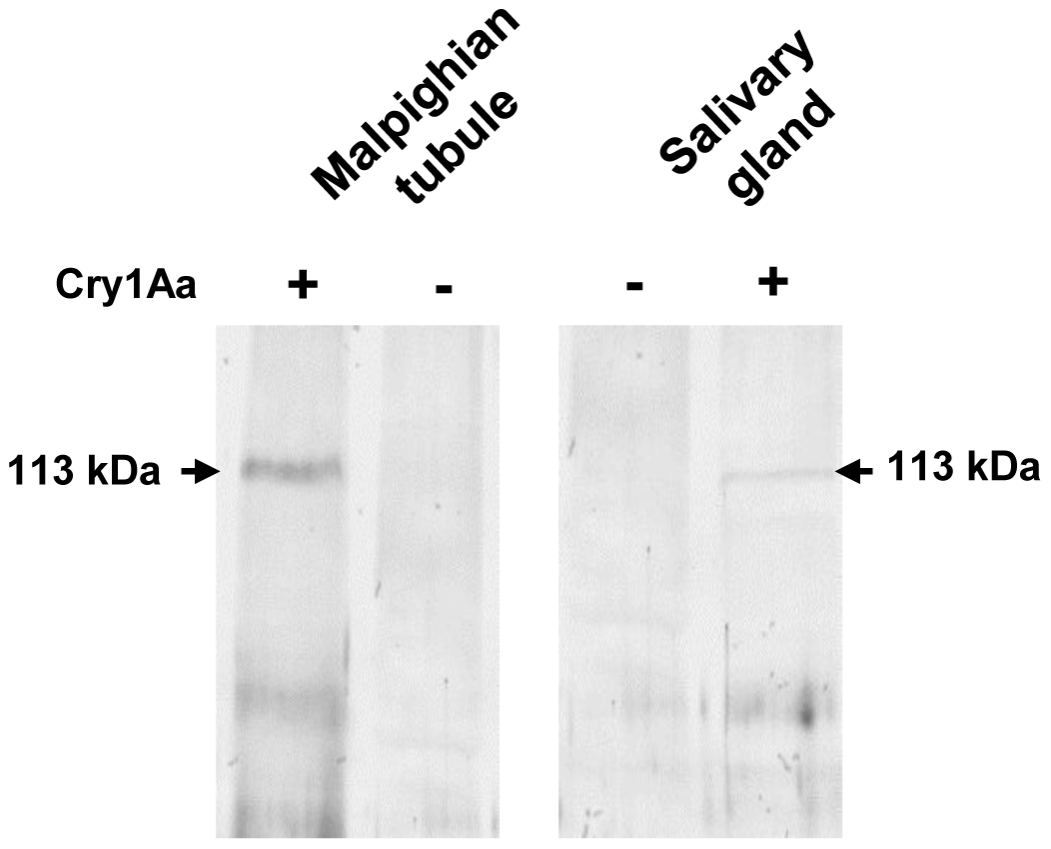
Immunoprecipitation of Cry1Aa toxin interacting protein. Triton X-100 solubilized Malpighian tubule and salivary gland membrane protein fractions (200 µg each) prepared from early fifth instar (5E) larvae were separately incubated with purified activated Cry1Aa toxin (5 µg) followed by further incubation with Cry1Aa polyclonal antibody (2.5 µg). The Cry toxin-interacting protein complex was pull-down with Protein A agarose beads, resolved by 7.5% SDS-PAGE, transferred onto a nitrocellulose membrane, then incubated with *A. janata* fat body APN polyclonal antibody, followed by incubation with ALP-conjugated secondary antibody and finally developed with NBT-BCIP substrate. Note the detection of a 113 kDa interacting membrane protein in both the tissues. (**−**) and (+) indicate absence and presence of Cry1Aa toxin respectively during incubation.

The specificity of interaction between the 113 kDa membrane protein (Malpighian tubule and salivary gland) and Cry1Aa toxin was confirmed by co-immunoprecipitation. Analysis of the pull-down Cry1Aa toxin-binding protein complex by western blot using *A. janata* fat body APN polyclonal antibody [14] detected the 113 kDa interacting membrane protein in both Malpighian tubule (Figure 2, Lane:+Cry1Aa) and salivary gland (Figure 2, Lane:+Cry1Aa). In fat body, this interaction has already been demonstrated by our group (data presented in Figure 8A in [14]). The control experiments which were performed in the absence of Cry1Aa toxin failed to show the cross-reactivity with the aforesaid antibody in Malpighian tubule (Figure 2, Lane: - Cry1Aa) as well as salivary gland (Figure 2, Lane: - Cry1Aa).

### Generation of AjAPN1 3D Molecular Structure and its Comparison with *B. mori* Midgut APN Structure

Position-Specific Iterated-Basic Local Alignment Search Tool (PSI-BLAST) against Protein Data Bank (PDB) revealed tricorn interacting factor F3 from *Thermoplasma acidophilum* and human endoplasmic reticulum aminopeptidase-1 (Erap1) to have the best sequence identity with AjAPN1 (Genbank ABE02186). The sequence identity of AjAPN1 with tricorn interacting factor F3 and human Erap1 was 28% and 27% respectively (Figure S1). The 3D structure of *B. mori* midgut APN was built based on the crystal structure of the soluble domain of human Erap1. The sequence identity between *B. mori* midgut APN and human Erap1 was 28%. The characteristic details of the templates are given in table 1. The first 41 amino acid residues of AjAPN1 sequence did not show enough sequence homology with the templates and therefore were not considered in the model construction. The 3D structures of AjAPN1 and *B. mori* midgut APN were generated using MODELLER interfaced by EasyModeller. The refined models were then validated for their stereo-chemical quality as well as side chain environment and the results as listed in table 2 showed that the quality of the models was good. The Ramachandran plot of AjAPN1 model showed 721 residues (87.6%) to fall in the most favored region, 87 residues (10.6%) in the additionally allowed region, 9 residues (1.1%) in the generously allowed region and 6 residues (0.7%) in the disallowed region (Figure S2). In *B. mori* midgut APN model, only 1 residue (0.1%) was in the disallowed region while 705 residues (86.5%) were in the most favored region, 97 residues (11.9%) in the additionally allowed region and 12 residues (1.5%) in the generously allowed region (Figure S3). The ERRAT results showed that the overall quality factor of the proteins was good with scores of 89.96 and 84.87 for AjAPN1 and *B mori* midgut APN respectively.

**Table 1:** Characteristics of the templates.

PDB code	Resolution [Å]	R-Value	R-Free
1Z1W	2.70	0.223 (obs.)	0.295
3QNF	3.0	0.231 (obs.)	0.283

**Table 2:** Validation results of the modeled proteins.

PROTEIN	PROCHECK (Ramachandran plot)		ERRAT
	allowed %	disallowed %	
AjAPN1	87.6	0.7	89.963
BmAPN	86.5	0.1	84.871

AjAPN1 represents non-gut hemocoelic tissue APN isoform of *A. janata* and BmAPN represents midgut APN of *B. mori*.

**Figure 3:**
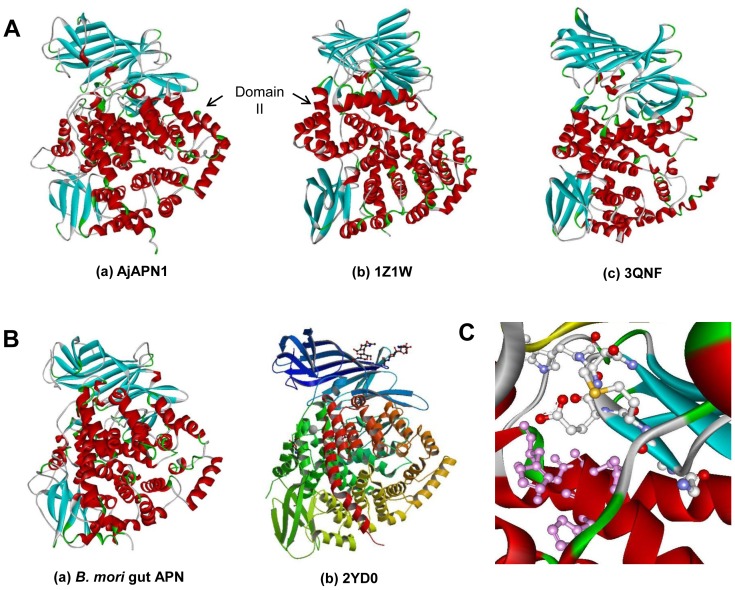
3D structures. (A) (a) AjAPN1 (model) and (b) tricorn interacting factor F3 (PDB code: 1Z1W) and (c) human endoplasmic reticulum aminopeptidase-1 (Erap1) (PDB code: 3QNF) (templates). (B) (a) *B. mori* midgut APN (model) and (b) soluble domain of human Erap1 (PDB code: 2YD0) (template). (C) Structure of APN activity and Zn^++^ binding motifs of AjAPN1. The important residues are shown in ball and stick. The APN catalytic amino acid residues are shown in white backbone while Zn^++^ binding amino acid residues are highlighted in pink.

The 3D structure of AjAPN1 was compared with that of *B. mori* midgut APN to find out if the Cry1Aa toxin binding region is structurally conserved. The overall fold of the two APN structures was found to be conserved (Figures 3Aa and 3Ba). The structures of aminopeptidase activity and Zn^++^ binding motifs of AjAPN1 (located in domain II) showing high similarity with *B. mori* midgut APN and templates are shown in figure 3C.

**Figure 4:**
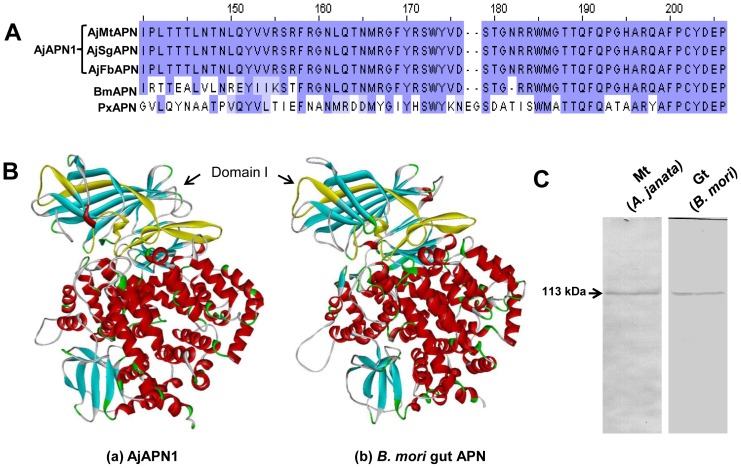
Analyses of Cry1Aa toxin interaction with AjAPN1. (A) Comparison of Cry1Aa toxin binding region of *B. mori* midgut APN (BmAPN) and *P. xylostella* midgut APN (PxAPN) with AjAPN1. (B) 3D structures of AjAPN1 and *B. mori* midgut APN. The secondary structure of the Cry1Aa toxin binding region located in domain I is highlighted in yellow. (C) *In vitro* Cry1Aa toxin binding analysis. Note the binding of Cry1Aa toxin to a 113 kDa membrane protein of Malpighian tubule of *A. janata*. Blot showing binding of Cry1Aa toxin to the 113 kDa protein of *B. mori* midgut BBMV act as a control. Mt: Malpighian tubule, Gt: gut, AjMtAPN: *A. janata* Malpighian tubule APN, AjFbAPN: *A. janata* fat body APN and AjSgAPN: *A. janata* salivary gland APN.

Amino acid sequences of AjAPN1, *B. mori* midgut APN and *Plutella xylostella* midgut APN (Genbank AAF01259) were aligned and the sequence identity in the Cry1Aa toxin binding region was compared. Multiple sequence alignment of the 64 amino acid residues of Cry1Aa toxin binding region is represented in figure 4A. In this region, the sequence identity of AjAPN1 with *B. mori* midgut APN and *P. xylostella* midgut APN was 81% (52 amino acid residues) and 48% (31 amino acid residues) respectively, which included the 27 amino acid residues common to both *B. mori* midgut APN and *P. xylostella* midgut APN (Figure 4A). The Cry1Aa toxin binding region located in domain I is highlighted in yellow (Figures 4Ba and 4Bb). The structures of Cry1Aa toxin binding regions of AjAPN1 and *B. mori* midgut APN as highlighted in yellow were highly similar to each other (Figure 4B). This structural similarity was further substantiated by *in vitro* binding of Cry1Aa toxin to the 113 kDa membrane protein of Malpighian tubule of *A. janata* where AjAPN1 of theoretical molecular weight of 111 kDa is expressed (Figure 4C). The blot showing binding of Cry1Aa toxin to midgut brush border membrane vesicles (BBMVs) protein of *B. mori* was used as a positive control (Figure 4C).

### Analyses of *AjAPN1* Transcript and its Encoded Protein Expression after Double-stranded siRNA Injection

**Figure 5:**
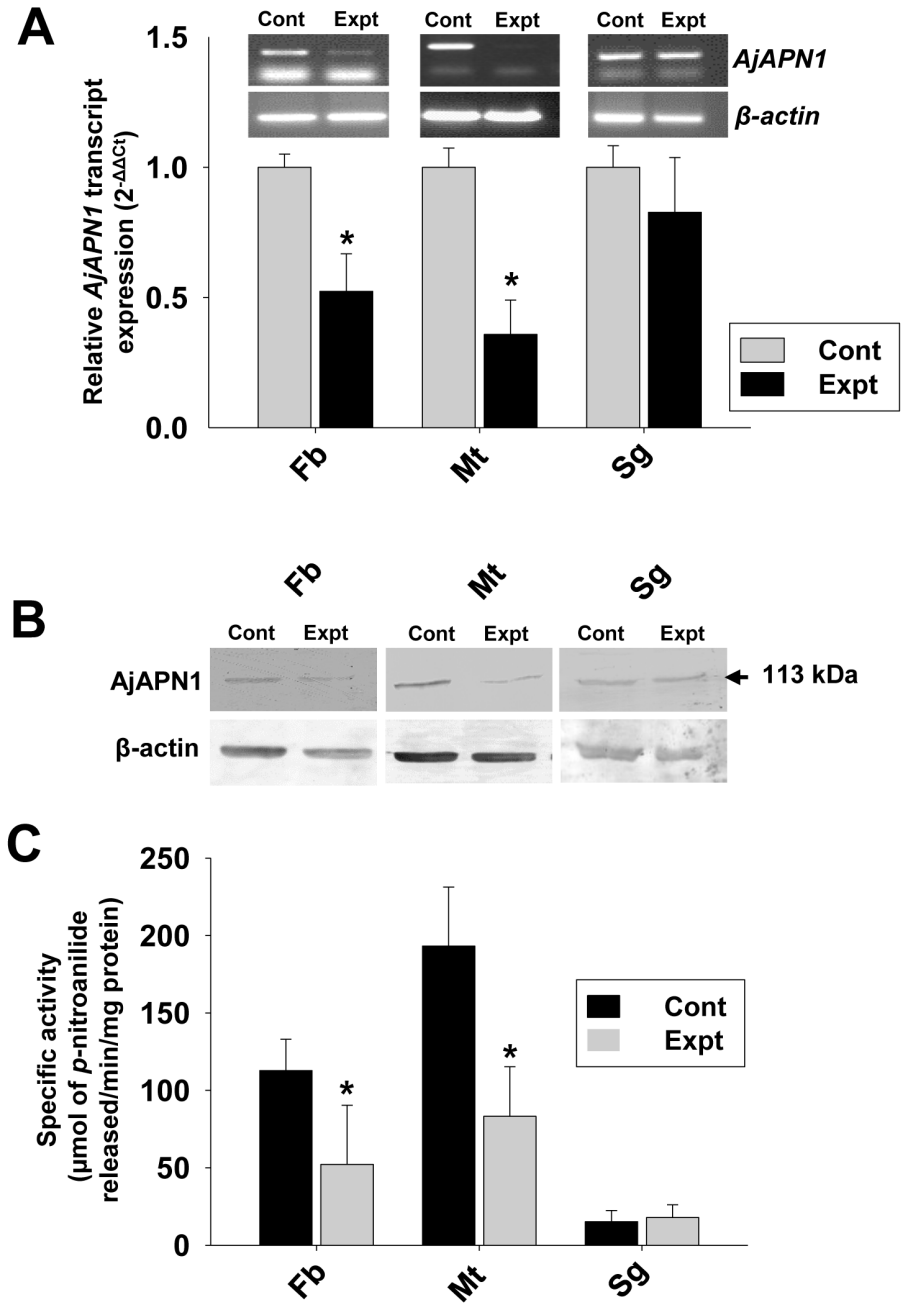
RNAi-mediated knockdown of *AjAPN1* transcript and its encoded protein. Third instar larvae were intrahemocoelically injected with target and control gene siRNA duplexes at dose of 5 µg/100 mg body weight followed by analyses of target gene/protein expression level at different time points. Observations obtained at 66 h post-injection are represented. Values represented are mean±standard deviation of three independent experiments (n = 3). Significance between groups was tested by One-Way ANOVA followed by Student-Newman-Keuls’ (SNK) test using SigmaPlot 11.0 software. *indicate statistical significance (P<0.05). Control (Cont): double-stranded *GFP* siRNA injected and Experimental (Expt): double-stranded *AjAPN1* siRNA injected insects. (A) Real-time quantitative PCR analysis. 18S rRNA was used as an internal endogenous control. Note that the fold decrease in *AjAPN1* transcript level in fat body and Malpighian tubule was 1.9 and 2.8 respectively. Semi-quantitative analysis is represented by the gel images. Here, *β*-actin gene was used as an internal endogenous control (lower panel). (B) Western blot analysis. Note the substantial reduction in the 113 kDa AjAPN1 protein band of fat body and Malpighian tubule of the target gene siRNA injected insects. β-actin expression was used as an internal endogenous control (lower panel). (C) APN activity analysis. Note the significant decrease in the APN activity level of fat body and Malpighian tubule of the target gene siRNA injected insects. Fb: fat body, Mt: Malpighian tubule and Sg: salivary gland.

Semi-quantitative and real-time quantitative PCR analyses of *AjAPN1* transcript level in the target gene siRNA injected third instar larvae showed substantial decrease in fat body and Malpighian tubule at 66 h post-injection (Figure 5A). However, the decrease in salivary gland was not significant (Figure 5A). The fold decrease in *AjAPN1* transcript level as quantified by real-time PCR was a significant (P<0.05) 1.9 and 2.8 in fat body and Malpighian tubule respectively. Correspondingly, western blot analysis showed a substantially reduced expression level of the target protein in fat body and Malpighian tubule of target siRNA injected insects as compared to the control insects (Figure 5B). The reductions in the APN activity levels of the target siRNA injected larvae as compared to the controls (injected with double-stranded *GFP* siRNA) were a significant (P<0.05) 50% and 56% for fat body and Malpighian tubule respectively (Figure 5C). The control larvae showed no significant changes in the *AjAPN1* transcript, protein as well as activity levels (Figures 5A, 5B and 5C).

### Effects of *AjAPN1* Gene Silencing on *A. janata* Development

**Figure 6:**
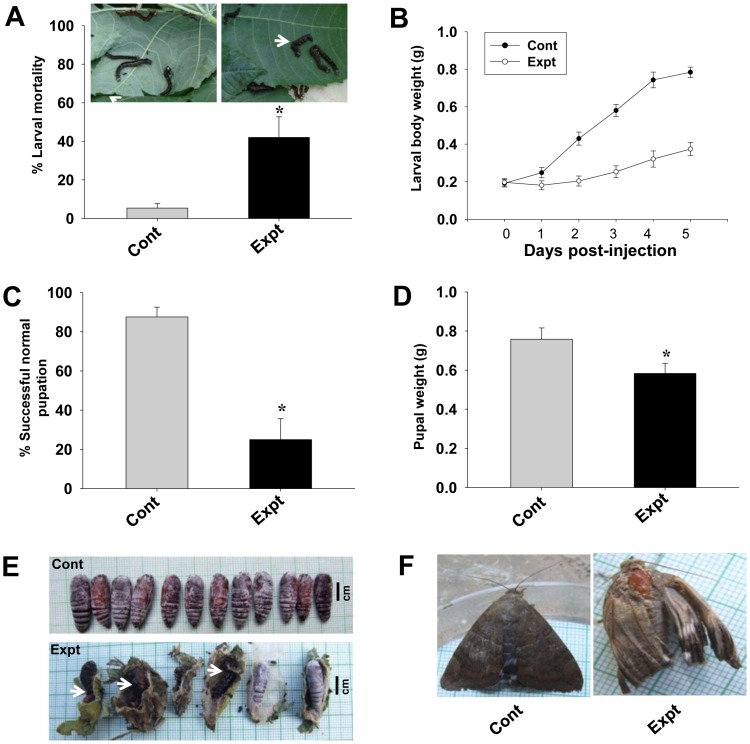
Effects of AjAPN1 RNAi on *A. janata* development. Third instar larvae were intrahemocoelically injected with target and control gene siRNA duplexes at dose of 5 µg/100 mg body weight. The larval growth rate was monitored by recording the weight of each larva every 24 h for 5 days. Larval mortality was calculated 2 day post-injection. Percentage of successful pupation and pupal weights were compared between the control and experimental groups. Values represented are mean±standard deviation of three independent experiments (n = 3). Significance between groups was tested by One-Way ANOVA followed by SNK test using SigmaPlot 11.0 software. *indicate statistical significance (P<0.05). (A) Larval mortality. Arrow indicates non-feeding inactive/death larvae. (B) Inhibition of larval growth. (C) Percentage of successful normal pupation. (D) Pupal weight. (E) Development of lethal larval-pupal intermediates. (F) Emergence of viable defective adults.

The larval mortality calculated 2 day post-injection revealed a significant (P<0.05) 42% larval deaths in the target gene siRNA injected insects, while in the control group, it was only 5% (Figure 6A). We observed a drastic inhibition of larval growth rate in the experimental group. Control larvae fed voraciously and grew normally throughout the larval stages, with each larva weighing approximately 0.78±0.03 g at fifth instar i.e., 5 day post-injection. On the other hand, all the target gene siRNA injected larvae either did not feed or fed less and hence showed arrested growth. Even at 5 day post-injection, each larva still weighed only 0.37±0.04 g (Figure 6B). Feeding inhibition and the subsequent larval growth arrest delayed pupation by 4–5 days in experimental insects (data not shown). Of the surviving larvae from the experimental group, only 25% (P<0.05) were able to undergo successful normal pupation, while 87% of the larvae from the control group successfully molted into normal pupae (Figure 6C). The pupae of the experimental group were fairly small with each pupa weighing only 0.58±0.05 g (P<0.05), while the control pupae were larger in size with each pupa weighing 0.75±0.03 g (Figure 6D). In addition, a significant percentage (P<0.05) of the experimental insects could not complete the larval-pupal transformation and died as “larval-pupal intermediates” exhibiting both larval as well as pupal phenotypes (Figure 6E, white arrow). The adults which emerged from the experimental group were found to be either smaller in size and/or phenotypically defective (Figure 6F). The fertility of the adults from the experimental group and fecundity of their eggs were also highly reduced (data not shown).

### Effect of *AjAPN1* Gene Silencing on Interaction with Cry1Aa Toxin

**Figure 7:**
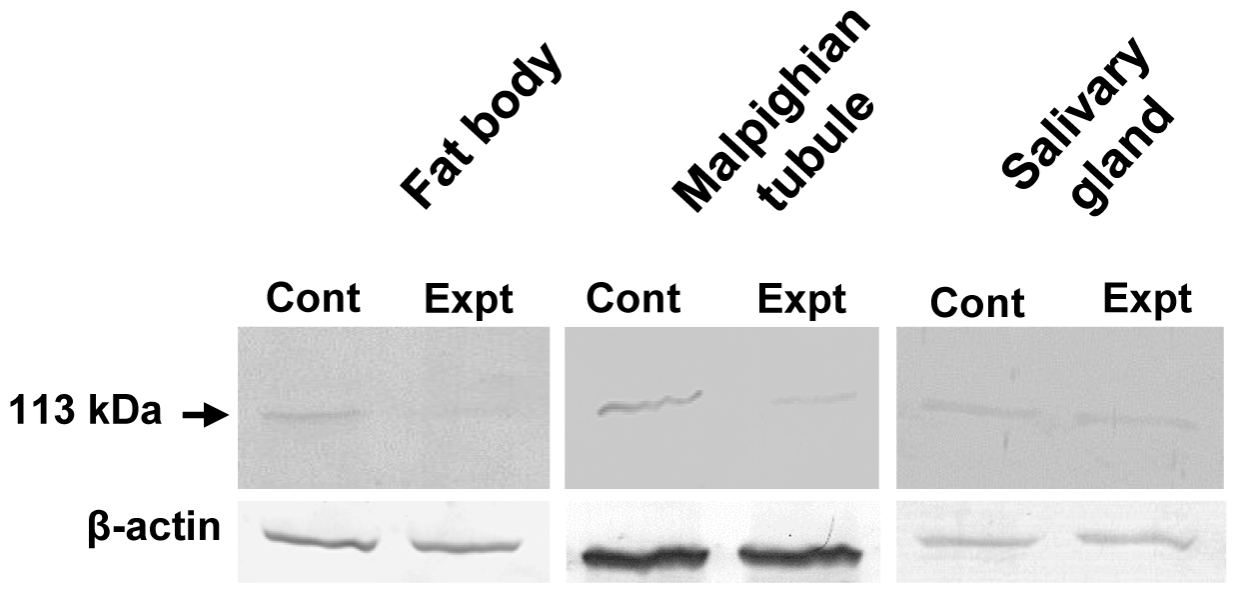
*In vitro* Cry1Aa toxin interaction in AjAPN1 knockdown larvae. Fat body (Fb), Malpighian tubule (Mt) and salivary gland (Sg) membrane protein fractions (30 µg each) of target and control gene siRNA injected larvae were separated by 7.5% SDS-PAGE, transferred onto a nitrocellulose membrane, then incubated with biotinylated Cry1Aa toxin (200 ng/mL) followed by further incubation with streptavidin-ALP conjugate (1∶1000 dilutions) and finally developed with NBT-BCIP substrate. Note the substantially reduced interaction of Cry1Aa toxin to the 113 kDa membrane protein of fat body and Malpighian tubule of the target gene knockdown larvae. Lower panels in each blot (β-actin expression) represent equal loading of proteins. Control (Cont): double-stranded *GFP* siRNA injected insects, Experimental (Expt): double-stranded *AjAPN1* siRNA injected insects.

*In vitro* Cry1Aa toxin binding analysis revealed a drastically reduced interaction of Cry1Aa toxin with the 113 kDa membrane protein of fat body and Malpighian tubule of the AjAPN1 knockdown insects (Figure 7). However, there was no difference in the intensity of interaction of Cry1Aa toxin with the 113 kDa membrane protein of salivary gland between the experimental and the control insects (Figure 7).

## Discussion

With the ever increasing world population and pronounced toxicological effects of chemical pesticides, the need for more effective and eco-friendly insect pest control approach in agriculture has become more urgent. Developing alternate approaches that could target novel molecules of non-gut hemocoelic tissues along with known molecular targets of the gut would not only facilitate effective management of insect pests but would also help in tackling the problem of increasing pests resistance to *B. thuringiensis* Cry toxins. Specific binding of Cry toxins to receptors of midgut epithelial cells is critical for induction of toxicity to the susceptible insects [26]–[28]. Midgut APNs as Cry toxin receptors have been well documented in a number of lepidopteran insects [5], [29]. Thus, Cry toxin-induced toxicity after oral ingestion follows a well-accepted mechanism of action. Interestingly, Cerstiaens *et al* first time demonstrated Cry toxin toxicity by hemocoelic delivery in *Lymantria dispar* (Lepidoptera) and *Neobellieria bullata* (Diptera) [30]. Earlier study in our laboratory also demonstrated binding of Cry toxins to larval fat body membrane protein of *A. janata* [14]. In the present study, we showed *in vitro* binding of Cry1Aa toxin predominantly to a 113 kDa membrane protein not only in fat body but also in Malpighian tubule and salivary gland of *A. janata*. Co-immunoprecipitation analysis demonstrated that the interaction was specific and further; using *A. janata* fat body APN polyclonal antibody, we indicated that the 113 kDa interacting membrane protein of these tissues could possibly be an APN. Therefore, this *in vitro* Cry1Aa toxin-113 kDa membrane protein interaction in these tissues could very well represent Cry toxin-APN interaction under *in vivo* condition. Further in the larval hemocoel, AjAPN1 expressing fat body, Malpighian tubules and salivary glands are bathed in hemolymph, which would likely facilitate the interaction of Cry1Aa toxin with the AjAPN1 protein associated with these tissues.

In *B. mori*, high susceptibility to Cry1Aa toxin and low susceptibility to Cry1Ac toxin was shown to be associated with high and low binding specificities to APN respectively [31]. *M. sexta* APN binds to Cry1Aa and Cry1Ac with equally high affinities and subsequently, the larvae were found to be highly sensitive to both Cry1Aa toxin and Cry1Ac toxin [32]. The binding regions for Cry1Aa and Cry1Ac in *B. mori* midgut APN were shown to be different [33], thus suggesting that different Cry toxin types could have different recognition region on APN. Yaoi *et al* identified the region between 135-Ile and 198-Pro in *B. mori* midgut APN as the Cry1Aa toxin binding region [33]. Comparison of this 64 amino acid residue Cry1Aa toxin binding region of *B. mori* midgut APN [33] and *P. xylostella* midgut APN [34] with AjAPN1 sequence revealed 81% and 48% amino acid sequence identity respectively. Of the 64 amino acid residues, 27 amino acid residues common to both *B. mori* and *P. xylostella* midgut APNs were suggested to be important for Cry1Aa toxin binding [33], [34]. Between AjAPN1 and *B. mori* midgut APN, 52 amino acid residues including the vital 27 amino acid residues were identical. AjAPN1 and *P. xylostella* midgut APN also shared 31 amino acid residues which also included the important 27 amino acid residues. Hence we assume the interaction between Cry1Aa toxin and 113 kDa membrane protein of fat body, Malpighian tubule and salivary gland of *A. janata* to be mediated by the presence of Cry1Aa toxin binding region in AjAPN1. In our earlier study, we demonstrated high Cry1A toxicity upon oral ingestion in *A. janata* and subsequently, the identification of Cry1A toxin binding region in the midgut APN of this species further strengthened this notion [14]. Thus, Cry toxins probably bind to a conserved region of an APN class which is specific to the type of Cry toxins, and not to all the APN classes. For instance, Cry1Aa toxin did not bind to 120 kDa APN of *L. dispar* BBMV [35]. However, it is more important to confirm whether AjAPN1 is merely a binding protein or a protein that can confer toxicity upon binding since binding of Cry toxins to midgut of non-susceptible insects has also been reported [36], [37].

It is reported that different Cry1A toxins have similar primary sequences as well as 3D structures and therefore recognize APNs with similar structure; the specificity of interaction being mediated by the structure of Cry1A toxin binding region of APN [38]. To provide better insights into the functional role of AjAPN1 towards understanding of Cry toxin-receptor interaction and its likely implications, we constructed a 3D molecular structure of AjAPN1 based on sequence identity and similarity with the templates. Since the sequence homology between the target and templates was low (<30%) and taking into consideration the varying degrees of sequence similarity that could exist between different regions of the target sequence and different templates [39], multiple templates rather than a single template was used. Despite low sequence identity and similarity, the model generated was reasonably good, primarily could be due to the conservation of residues corresponding to the APN signature motifs, position specific residues and stereo specific residues as they were all taken into account during the construction of the model. Cry1Aa-binding *B. mori* midgut APN 3D model was also constructed for comparison. The overall folding pattern of AjAPN1 and *B. mori* midgut APN 3D structures was highly conserved with the available 3D structures of the experimentally determined gluzincin aminopeptidases. This suggests that the structure of different APNs share a conserved conformation but the Cry toxin recognition or binding site could vary. Interestingly, AjAPN1 and *B. mori* midgut APN shared high sequence and structural similarity in the Cry1Aa toxin binding region, and therefore it is highly probable that both the APNs are Cry1Aa toxin receptors. Besides, the ability of Cry1 toxins to easily induce toxicity in a broad spectrum of insect pests by recognizing the conserved structures of APNs further strengthened the present case [34], [40]. In the present study, the potential functional role of AjAPN1 as a Cry1Aa toxin receptor was further supported by *in vitro* interaction of Cry1Aa toxin to the 113 kDa membrane protein of Malpighian tubule where AjAPN1 of theoretical molecular weight of 111 kDa was expressed.

To further substantiate AjAPN1 as a potential Cry1Aa toxin receptor in non-gut hemocoelic tissues of *A. janata*, we employed RNAi approach for silencing the expression of the protein. Recently, Terenius *et al* suggested that either for feeding or hemocoelic injection, fairly high concentrations of dsRNA are required to achieve high degree of silencing in lepidopteran insects [41]. In *H. armigera*, feeding of short dsRNAs rather than long dsRNA produced higher level of silencing [42]. Studies also showed that short dsRNAs have had the most success when delivered into the insect hemocoel by microinjection rather than by feeding [43]–[45]. Furthermore, the alkaline pH and the numerous RNases present in the lepidopteran gut are known to provide hostile environment for RNAs [46]. Hence for the present study, we opted to inject relatively high doses of double-stranded siRNAs into the larval hemocoel. Injection of *AjAPN1* siRNA duplexes at concentrations of 1, 2.5 and 5 µg/100 mg body weight either to fourth or fifth instar larvae did not yield any desired result. When fourth and fifth instar larvae were used, we found that there was a minor decline in *AjAPN1* transcript levels in all the three tissues at 3–4 day post-injection, which possibly could be due to normal developmental regulation of the gene rather than the consequence of RNAi. Earlier reports showed that hemocoelic injection of dsRNA to fifth instar larvae of *S. litura* [13] and *H. armigera* [21] resulted in high degree of APN gene silencing but not in *Ostrinia nubilalis*, *Spodoptera exigua* and *Epiphyas postvittana* [41] and thus, the later report corroborated well with our observation in *A. janata* where *AjAPN1* gene silencing could not be achieved when target double-stranded siRNA was injected into fifth instar larvae. When third instar larvae were injected with 5 µg/100 mg body weight of target siRNA duplexes and analyzed at different time points, we observed a significant 1.9 and 2.8-fold decrease in *AjAPN1* transcript level of fat body and Malpighian tubule respectively at 66 h post-injection. Consequently, there was a corresponding substantial decrease in the AjAPN1 protein expression level and significant reductions of 50% and 56% in the APN activity levels of fat body and Malpighian tubule respectively. The control (*GFP* siRNA) used in the experiment further confirmed that the responses were specific to the gene for which it was employed. Our results demonstrated that the susceptibility of an insect species to siRNA could also depend on the nature of the tissue and larval stage of development. This observation is not surprising as earlier studies have shown that regardless of the delivery methods, RNAi effects may vary in different species, instars or even among individuals within the same species of lepidopteran insects [47], [48].

High percentage of larval mortality as a consequence of target siRNA injection indicated that *AjAPN1* gene silencing may be lethal to *A. janata* development. The larval growth arrest, development of lethal larval-pupal intermediates, development of smaller pupae and emergence of viable defective adults as a result of *AjAPN1* gene silencing clearly indicated that this gene product plays important physiological role(s) during post-embryonic development and metamorphosis of *A. janata*. In *Diatraea saccharalis*, only a partial suppression of APN expression was enough to bring about significant decrease in Cry1Ab susceptibility [49]. In the present study, as injection of target siRNA to third instar larvae resulted in high mortality rate, Cry1Aa toxin hemocoelic delivery-based larval toxicity assay conducted on AjAPN1 knockdown larvae was not feasible. Hence we performed *in vitro* Cry1Aa toxin binding assay which revealed a drastically reduced interaction of Cry1Aa toxin with the 113 kDa membrane protein of fat body and Malpighian tubule of the AjAPN1 knockdown larvae correlating well with significantly inhibited expression of the target gene. These results further demonstrated that the interaction of Cry1Aa toxin with the 113 kDa membrane protein of non-gut hemocoelic tissues of *A. janata* is specific.

In conclusion, findings from the present study suggest AjAPN1 expression in non-gut hemocoelic tissues to play important physiological role(s) during post-embryonic development of *A. janata*. Secondly, we demonstrated specific interaction of Cry1Aa toxin with the 113 kDa AjAPN1 protein of non-gut hemocoelic tissues and indicated its potential role as a Cry1Aa toxin receptor in these tissues of *A. janata*. However, its functional role as a Cry1Aa toxin receptor in *A. janata* still remains to be proved.

## Materials and Methods

### Insect Culture

*A. janata* neonate larvae were obtained from Directorate of Oilseeds Research, Hyderabad, India and reared on fresh castor leaves (*Ricinus communis*) as diet under photoperiod of 14∶10 h (light:dark) and 60–70% relative humidity at 27±2°C in the insect culture facility of our laboratory. The larval development proceeds through five instars and the whole life cycle is completed in about 45–50 days. Each instar from first to fourth lasts for 2 days while the last or fifth instar lasts 4–5 days. The fifth instar is further classified into early (5E) and late (5L) fifth instar which lasts 2 and 2–3 days respectively, then followed by non-feeding pre-pupal (PP) stage. For RNAi studies, third, fourth and 5E instars while for all other studies, only 5E instar larvae were used. Tissues were dissected out in ice-cold insect Ringer solution (130 mM NaCl, 0.5 mM KCl, 0.1 mM CaCl_2_) and used immediately.

### Purification and Biotinylation of Activated Cry1Aa Toxin

Cry1Aa protoxin was prepared from recombinant *Escherichia coli* JM103 strain ECE52 harboring *cry1Aa* gene [50], which was supplied by *Bacillus* Genetic Stock Centre (Ohio State University, USA). The protoxin was activated [50], purified by gel filtration on Sephadex G-100 column and biotinylated using a kit (Bangalore Genei).

### *In vitro* Cry1Aa Toxin Binding Analysis

Brush border membrane vesicles (BBMVs) of midgut were prepared according to the method described by Wolfersberger *et al* [51]. Fat body, Malpighian tubule and salivary gland membrane protein fractions were prepared as described by Kirankumar *et al* [52]. The protein samples were separated by 7.5% SDS-PAGE, electro-transferred onto nitrocellulose membranes (Pall Life Sciences) and blocked in a blocking buffer [3% BSA (w/v) in 0.01 M Tris-buffered saline (TBS, pH 7.4)] for 1 h. The blots were first incubated in the same buffer containing biotinylated Cry1Aa toxin (200 ng/mL) for 1 h, followed by incubation for additional 2 h in the same buffer containing streptavidin-alkaline phosphatase (ALP) conjugate (1∶1000 dilutions) and finally developed with NBT-BCIP substrate (Sigma-Aldrich). The blots incubated with the same concentration of the unlabeled Cry1Aa toxin were used as controls.

### Immunoprecipitation of Cry1Aa Toxin Interacting Proteins

Triton X-100 solubilized Malpighian tubule and salivary gland membrane protein preparations (200 µg each) were incubated with purified activated Cry1Aa toxin (5 µg) in binding buffer (50 mM sodium phosphate, pH 7.5, 50 mM NaCl and 3 mM MgCl_2_) for 12 h at 4°C. This was followed by addition of 5 µl of purified Cry1Aa polyclonal antibody (2.5 µg) and incubated for another 3 h. Subsequently, 50 µl of Protein A agarose beads was added to the mixture and incubated for another 2 h at 4°C on a rotary shaker. The agarose beads were pelleted, washed six times with binding buffer, re-suspended in equal volume of 2X SDS sample buffer containing 2-mercaptoethanol [125 mM Tris-HCl, pH 6.8; 4% (w/v) SDS; 20% (v/v) glycerol; 10% (v/v) 2-mercaptoethanol; 0.002% Bromophenol Blue] and heated at 100°C for 5 min. The pull-down Cry1Aa toxin-interacting proteins were separated by 7.5% SDS-PAGE, electro-blotted onto nitrocellulose membrane (Pall Life Sciences), incubated with *A. janata* fat body APN polyclonal antibody (1∶10000 dilutions) [14], followed by incubation with ALP-conjugated goat anti-rabbit IgG and finally developed with NBT-BCIP substrate (Sigma-Aldrich).

### Construction of 3D Molecular Structures of AjAPN1 and *B. mori* midgut APN

The crystal structures of tricorn interacting factor F3 from *T. acidophilum* (PDB code: 1Z1W) [40] and human endoplasmic reticulum aminopeptidase-1 (Erap1) (PDB code: 3QNF) [53] were selected as template structures. The BLAST program [54] and PDB [55] available at National Centre for Biotechnology Information (NCBI) were used to select the template structures. Sequences corresponding to AjAPN1 of *A. janata* and midgut APN of *B. mori* were obtained from NCBI (http://www.ncbi.nih.gov). For model construction, we used MODELLER program [56] interfaced by EasyModeller [57] and twenty models were generated and analyzed. The model showing the best DOPE score was saved for further refinement and validation. Layers of water with thickness 10 Å were added to the whole protein using the VMD software [58]. The protein model was energy minimized using CHARMM forcefield of NAMD [59]. The energy minimization was carried out using 1000 steps of steepest descent followed by 10000 steps of conjugate gradient to relieve all the bad contacts of the system. The quality of the refined structure obtained was checked with ERRAT program [60]. The ERRAT program was used to assess the false statistics of bad non-bonded interactions within the model structure. The stereochemical quality of the model was examined by a Ramachandran plot using PROCHECK program [61] and Profile-3D programs [62]. The number of residues that are in the allowed or disallowed regions of the Ramachandran plot determines the quality of the model. The Profile-3D tests the validity of the hypothetical protein structure by measuring the compatibility of the structure with its own amino acid sequence. The root mean square deviation of the model with respect to Cα atoms of the template was measured using the Combinatorial Extension (CE) method [63]. The *B. mori* midgut APN 3D model was constructed using the crystal structure of the soluble domain of human Erap1 (PDB code: 2YD0) as the template.

### Preparation and Injection of Double-stranded Target and Control Gene siRNAs to *A. janata* Larvae

*AjAPN1* gene siRNA duplexes of 19-nt each of sense and antisense strands was custom-designed, synthesized and commercially supplied by Sigma-Aldrich (Figure S4). The siRNA duplex sequence comprised 5′CUCUUUCAAACAUGCCGAUdTdT3′ and 5′AUCGGCAUGUUUGAAAGAGdTdT3′ as a sense and antisense sequence respectively which targeted the specific region “CTCTTTCAAACATGCCGAT” of *AjAPN1* gene. For the control, *GFP* gene siRNA duplex comprising 5′GAACUUCAGGGUCAGCUUGCC3′ and 5′GCAAGCUGACCCUGAAGUUCA3′ as sense and antisense strands respectively were prepared as described by Donzé and Picard [64].

After narcotization on ice, third instar larvae were intrahemocoelically injected with siRNA duplexes at a dose of 5 µg/100 mg body weight through the dorsal side. Each experiment was performed with 18 larvae. Injection was carried out with a home-assembled microsyringe (Figure S5). A glass needle prepared with a micropipette puller (Model P-2000, Sutter Instruments Co. USA) was fitted to a Hamilton microsyringe holder through a sterile plastic tube. Care was taken to incur minimum injury and prevent hemolymph leakage. After injection, the wounds were sealed immediately with bee wax, placed again on ice for 10 min, then transferred back to the culture chamber and reared on fresh castor leaves. Tissues were dissected out at different time points in ice-cold insect Ringer solution and used for subsequent studies. Tissues from four larvae were pooled together for each sample.

### Semi-quantitative and Real-time PCR Analyses of *AjAPN1* Gene Silencing

Total RNAs were isolated using TRI® Reagent (Sigma-Aldrich) and then reverse transcribed to corresponding cDNAs using SuperScript III first strand synthesis kit (Invitrogen). For semi-quantitative PCR analysis, a primer pair of 5′TGGCTGGATATGGTATTACT3′ and 5′GATATTGAATGCTCTGGTGTA3′ was used as forward and reverse primer respectively to amplify a 476 bp fragment of *AjAPN1* gene. A primer pair of 5′GGTAGTAGACAATGGCTCGGG3′ and 5′CCCAGTTAGTGACGATTCCGTG3′ was used as forward and reverse primer respectively to amplify a 180 bp fragment of lepidopteran insect *β*- actin gene for use as an internal control.

Real-time PCR analysis was performed in a 20 µl reaction volume using a custom-made Taqman gene expression assay (Applied Biosystems). A forward primer 5′AGGAATACACAGGCTATCCGTACT3′, a reverse primer 5′GGCTGCTTGCTGCATGAT3′ and a Taqman probe “CAATGACCGAGAACATC” were used for the analysis of *AjAPN1* transcript levels. Insect 18S rRNA (Applied Biosystems) was used as an internal reference to normalize the *AjAPN1* transcript levels. *AjAPN1* transcript levels of the target siRNA injected larvae are presented as change in the transcript levels relative to the control siRNA injected larvae using the 2^−ΔΔCt^ method described by Livak and Schmittgen [65]. Applied Biosystems 7500 Fast Real-Time PCR system was used.

### Western and Activity Analyses after *AjAPN1* Gene Silencing

The membrane protein fractions of fat body, Malpighian tubule and salivary gland from target and control gene siRNA injected larvae were electrophoretically separated by 7.5% SDS-PAGE and electro-blotted onto nitrocellulose membrane (Pall Life Sciences) [66]. Non-specific binding sites were blocked with 5% skimmed milk (w/v) in TBS and then incubated with *A. janata* fat body APN polyclonal antibody (1∶10000 dilutions) [14]. Subsequently, the blots were incubated with ALP-conjugated goat anti-rabbit IgG (Bangalore Genei) and developed with NBT-BCIP substrate (Sigma-Aldrich). APN activity was determined according to the method described by Garczynski and Adang [2]. The molar absorbance co-efficient of *p*-nitroanilide was taken as 9.9×10^−3^ mol/L [67]. The specific activity was expressed as µmol of *p*-nitroanilide released/min/mg protein.

### Evaluation of Phenotypic Features after *AjAPN1* Gene Silencing

The phenotypic differences between target and control gene siRNA injected insect groups were investigated by examining larval mortality, larval growth rate, pupal weights and analysis of larval-pupal as well as pupal-adult transformations. The larval weight was recorded every 24 h for 5 days. Larval mortality was calculated 2 day post-injection. Phenotypic features were analyzed every 2 day until all adults eclosed. Photographs were taken with FinePix S9600 digital camera (Nikon).

### Statistical Analysis

Data are expressed as mean±standard deviation of three independent experiments (n = 3). Differences between groups were analyzed for statistical significance by One-Way ANOVA followed by Students-Newman-Keuls’ (SNK) test using SigmaPlot 11.0 software (Systat Software Inc., USA). A probability of P<0.05 was considered statistically significant.

## Supporting Information

Figure S1 **Sequence alignment.** AjAPN1 amino acid sequence was alligned with tricorn interacting factor F3 from *Thermoplasma acidophilum* (PDB code: 1Z1W) and human endoplasmic reticulum aminopeptidase-1 (Erap1) (PDB code: 3QNF) templates using S-alignment software (MODELLER).
(DOC)

Figure S2 **Ramachandran plot of AjAPN1 molecular model.** Amino acid residues in red, yellow, cream and white shaded regions are in most favorable, additionally allowed, generously allowed and disallowed regions, respectively.
(DOC)

Figure S3 **Ramachandran plot of** ***B. mori*** **midgut APN molecular model.** Amino acid residues in red, yellow, cream and white shaded regions are in most favorable, additionally allowed, generously allowed and disallowed regions, respectively.
(DOC)

Figure S4 **Integrity of double-stranded** ***AjAPN1*** **siRNA oligonucleotides.** Analysis of integrity of the double-stranded siRNA duplexes was performed by non-denaturing polyacrylamide gel electrophoresis and visualized by UV shadowing. Lanes M: oligonucleotide marker, Lanes 1 and 2: double-stranded *AjAPN1* siRNA duplexes.
(DOC)

Figure S5 **Home-assembled microsyringe set-up.** Hamilton microsyringe holder was fitted to a glass needle through a sterile plastic tube. The glass needles were prepared using a micropipette puller (Model P-2000, Sutter Instruments Co. USA).
(DOC)
