# Supplementary material for: Functional Interpretation of a Non-Gut Hemocoelic Tissue Aminopeptidase N (APN) in a Lepidopteran Insect Pest Achaea janata
Source: PLoS One. 2013 Nov 14;8(11):e79468. doi: 10.1371/journal.pone.0079468 (PMC3828369; doi:10.1371/journal.pone.0079468)
Supplement: Figure S1 — Sequence alignment. AjAPN1 amino acid sequence was alligned with tricorn interacting factor F3 from Thermoplasma acidophilum (PDB code: 1Z1W) and human endoplasmic reticulum aminopeptidase-1 (Erap1) (PDB code: 3QNF) templates using S-alignment software (MODELLER). (DOC) [file pone.0079468.s001.doc]

>P1;1Z1Wmono/1-780

1Z1Wmono 780 residues

---------------------------------------------------MEVEKYDLTLDFDIQK-----

----RTFNGTETITADAGDIVLDAVGLQINWMKVNG---RDTAFTYDGQTVRAPGDSQ--------------

-------PQKIEISFAGKVSDSLSGIY---YAGRENG---MITTHFEATDARRMFPCVDHPAYKAVFAITVV

IDKDYD-AISNMP-PKRIEVSERKVVE-FQDTPRMSTYLLYVGIGKF-RYEYEKYRDIDLILASLKDI--RS

KYPLDMARKSVEFYENYFGIPYALPKMHLI----SVPEFGAGAMENWGAITFRE--IYMDIAENSAVTVKRN

SANVIAHEIAHQWFGDLVTMKWWNDLWLNESFATFMSYKTMDTLFPEWSFWGDFFVSRTSG-ALRSDSLKNT

HPIEVDVRD-PDEISQIFDEISYGKGASILRMIEDYAGYEEFRKGISKYLNDHKFGNAEGSDLWTAIEDVS-

--------GKPVKRVMEYWIKNPGYPVIKLKR---NGRKITMYQTRFLLNGEEE--GRWPVPVNI--KKKDG

VE-------RILLEDEASI----EADGLIKINADSAGFYRVLYDDATFSDVMGHYR------DLSPLDRIGL

VDDLFAFLLSGHIDPETYRQRIRNFFDDEDHNV-----ITAIVGQMEYL-RMLTHAFD--DDARAFCRSRMQ

FLTGKQ---DENLKIALGRVSRLYVMV------DESYAEEMSKLFKDFDSAEPEMRSSIATAYALVTG----

---DLKGLLEKFRSVDRDEDRVRIISAFGKLKSNTDLS----TVYGMVEKTEIKKQDMISFFSSAL-----E

TLPGREFIFANLDRIIRLV----------------------IRYFTGNRTASRTVEM---------------

---------------------------MIPVIGLDHPDAEDIVRNIGSKNISMGLAKGIEMLAVNRKLVERI

RQTAVK--*

>P1;3QNFmono/1-696

3QNFmono 696 residues

----------------------PFPWNKI-----------------RLPEYVIPVHYDLLIHANLTT-----

----LTFWGTTKVEITASQPT-STIILHSHHLQISRATLRKGLSEEPLQVLEHPRQEQIALLA--------P

EPLLVGLPYTVVIHYAGNLSETFHGFYKSTYRTKEGELRILASTQFEPTAARMAFPCFDEPAFKASFSIKIR

REPRHL-AISNMPLVKSVTVAEGLIEDHFDVTVKMSTYLVAFIISDFESVSKITKSGVKVSVYAVPDKINQA

DYALDAAVTLLEFYEDYFSIPYPLPKQDLA----AIPDFQSGAMENWGLTTYRESALLFD-AEKSSASSKLG

ITMTVAHELAHQWFGNLVTMEWWNDLWLNEGFAKFMEFVSVSVTHPELKV-GDYFFGKCFD-AMEVDALNSS

HPVSTPVEN---------DDVSYDKGACILNMLREYLSADAFKSGIVQYLQKHSYKNTKNEDLWDSMASIV-

----------DVKTMMNTWTLQKGFPLITITV---RGRNVHMKQEHYMKTG-----YLWHVPLTFITSKSDM

VH-------RFLLKTKTDVLILPEEVEWIKFNVGMNGYYIVHYEDDGWDSLTGLLK--GTHTAVSSNDRASL

INNAFQLVSIGKLSIEK-ALDLSLYLKHETEIMPVFQGLNELIPMYKLMEKRDMNEVE--TQFKAFLIRLLR

DLIDKQTWTD-EG-SVSERMLRSQLLLLACVHNYQPCVQRAEGYFRKWKESQ--------IEFALC------

-----------------------------------------------------RTQNIEENIG---------

------WMDKNFDK---------------------------IRVWLQS------------------------

------------------------------------------------------------------------

--------*

>P1;AjAPN1/1-994

AjAPN1 994 residues

MASRWFNLLLGVLFIQGYLAFSPIPEERLMDEEWVEYNSMLRDPAYRLPTTTRPSHYAVTLTPYIESVPTGV

TADLFTFDGEATMTIQATEANVNEIRLHCNDLTILELTVH--VATDLTVNLATPGQTYECVMPWSFLTIPLT

TTLNTNLQYVVRSRFRGNLQTNMRGFYRSWYVDSTGNRRWMGTTQFQPGHARQAFPCYDEPGFKARFDITIV

RSPTFSPTLSNMPILSTTTLTNGWVAETFHTSTVTSTYLLAFIVSHYERVASSTDPERPFYIYARDNVGDTG

EWSLEIGEKLLLAMEEYTGYPYYSMTENIIMQQAAIPDFSAGAMENWGLLTYREALILYD-RLNSNHFYRQR

VANIVSHEIAHMWFGKLVTCAWWDNLWLNEGFARFYQYFLTDSVDETLGY-RTRFITEQLQVALLSDSEDSA

HALTNPAVNTPTTVSAHFSTITYAKGAAVLKMTQYLLGEKTYRRGLQSYLQANQYDVAEPEDLFSALDAAAV

VDNALAGYGITIEEYMKTWSEQAGHPLLSVSVDHSTGNMVVTQHRWNVNTGVSAISSLWHVPITW--TRAGA

VDFDNLKPTQILSGTATSINRGSTGREWVIFNKQQSGFYRVNYDSDTWALITQALRDSNSRTQIHEYNRAQI

VDDVFILARAGVLTYTR-AFNILSFLEFEDQYAP----WDAAITGFNFSRRRLAHNTESLEQLHALIYKLSE

AVTRRLGFAEIEGESYMDGLLRMYVNTFLCNVGHEECVQAGRTAFANWKNSGTFIPANM-RPWVYCTGLRYG

DASDFDFFWQQYLATDLASEQVVKLQAAGCTTDEASLGRYLDAITGGADDYQIRDQDIATALSSAITANEVN

TMRAFNWLTNNVERTTLALGSIQTPLSTITQRLLNTEQINTVSAWLQANSASLGTDIYNVGLSGIATSQNNI

AWYNQRISEYNSYFENGYIDETFEDEQTTPAATTEAPTTEADAGTTPDSASIATLSFVTLFVTLALNLAYKL

LSYIYDSL*
