# Supplementary figures and images for: Functional Interpretation of a Non-Gut Hemocoelic Tissue Aminopeptidase N (APN) in a Lepidopteran Insect Pest Achaea janata
Source: PLoS One. 2013 Nov 14;8(11):e79468. doi: 10.1371/journal.pone.0079468 (PMC3828369; doi:10.1371/journal.pone.0079468)

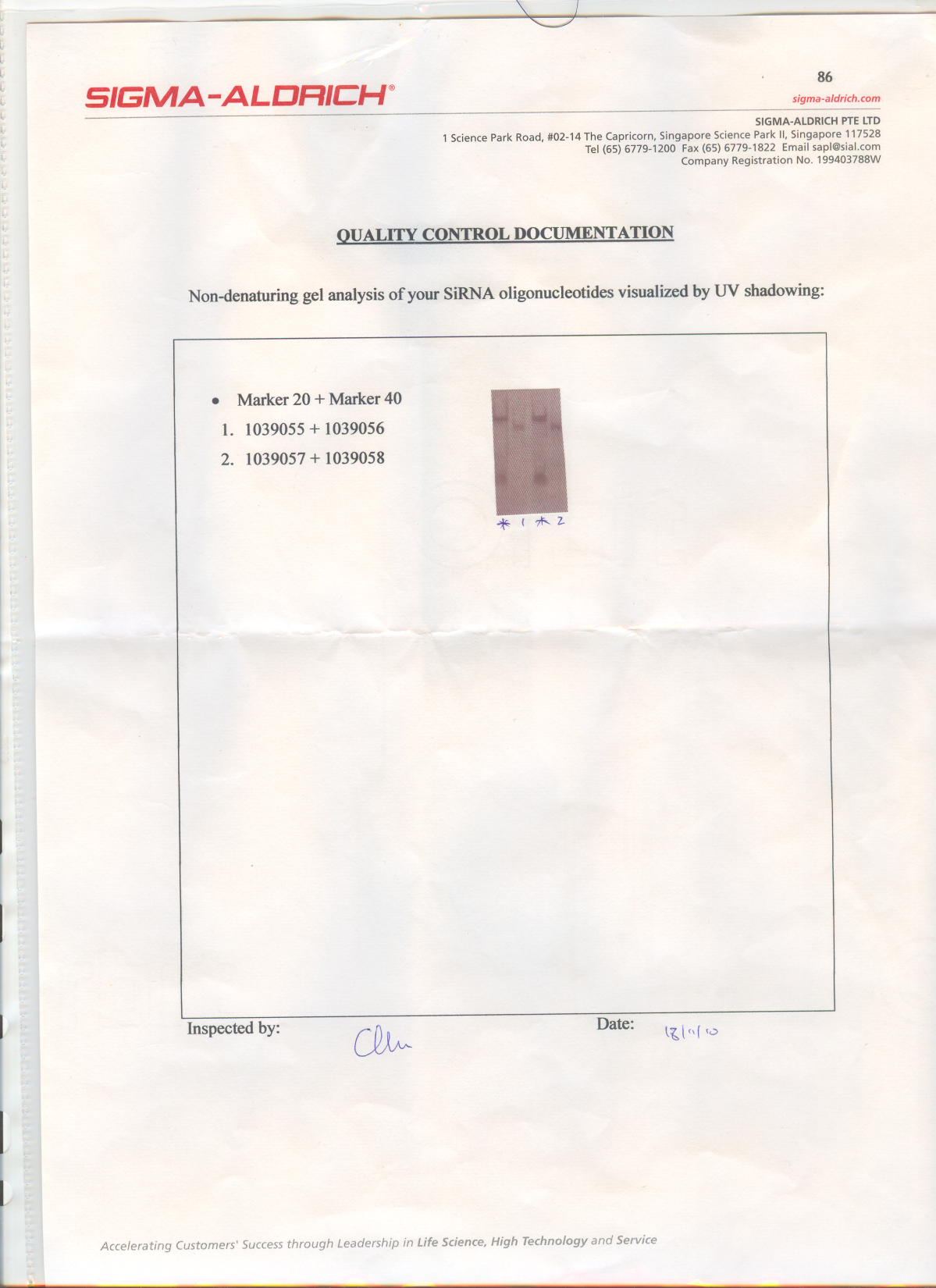


**M 1 M 2**

**40 mer**

**20 mer**

**Duplex siRNA**

**(19 oligomer)**

Supplement: Figure S4 — Integrity of double-stranded AjAPN1 siRNA oligonucleotides. Analysis of integrity of the double-stranded siRNA duplexes was performed by non-denaturing polyacrylamide gel electrophoresis and visualized by UV shadowing. Lanes M: oligonucleotide marker, Lanes 1 and 2: double-stranded AjAPN1 siRNA duplexes. (DOC) [file pone.0079468.s004.doc]

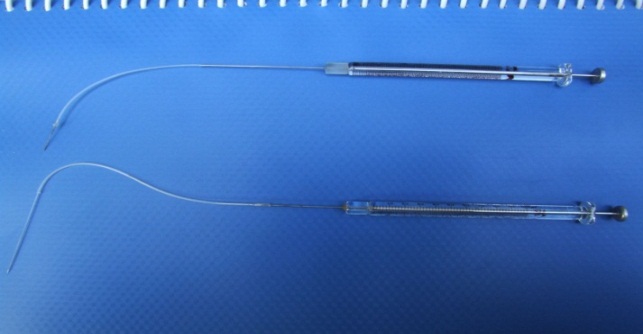


**Hamilton microsyringe holder**

**Glass needle**

**Plastic tube**

Supplement: Figure S5 — Home-assembled microsyringe set-up. Hamilton microsyringe holder was fitted to a glass needle through a sterile plastic tube. The glass needles were prepared using a micropipette puller (Model P-2000, Sutter Instruments Co. USA). (DOC) [file pone.0079468.s005.doc]
