# Supplementary material for: Functional Interpretation of a Non-Gut Hemocoelic Tissue Aminopeptidase N (APN) in a Lepidopteran Insect Pest Achaea janata
Source: PLoS One. 2013 Nov 14;8(11):e79468. doi: 10.1371/journal.pone.0079468 (PMC3828369; doi:10.1371/journal.pone.0079468)
Supplement: Supplementary file 4 [file pone.0079468.s003.doc]

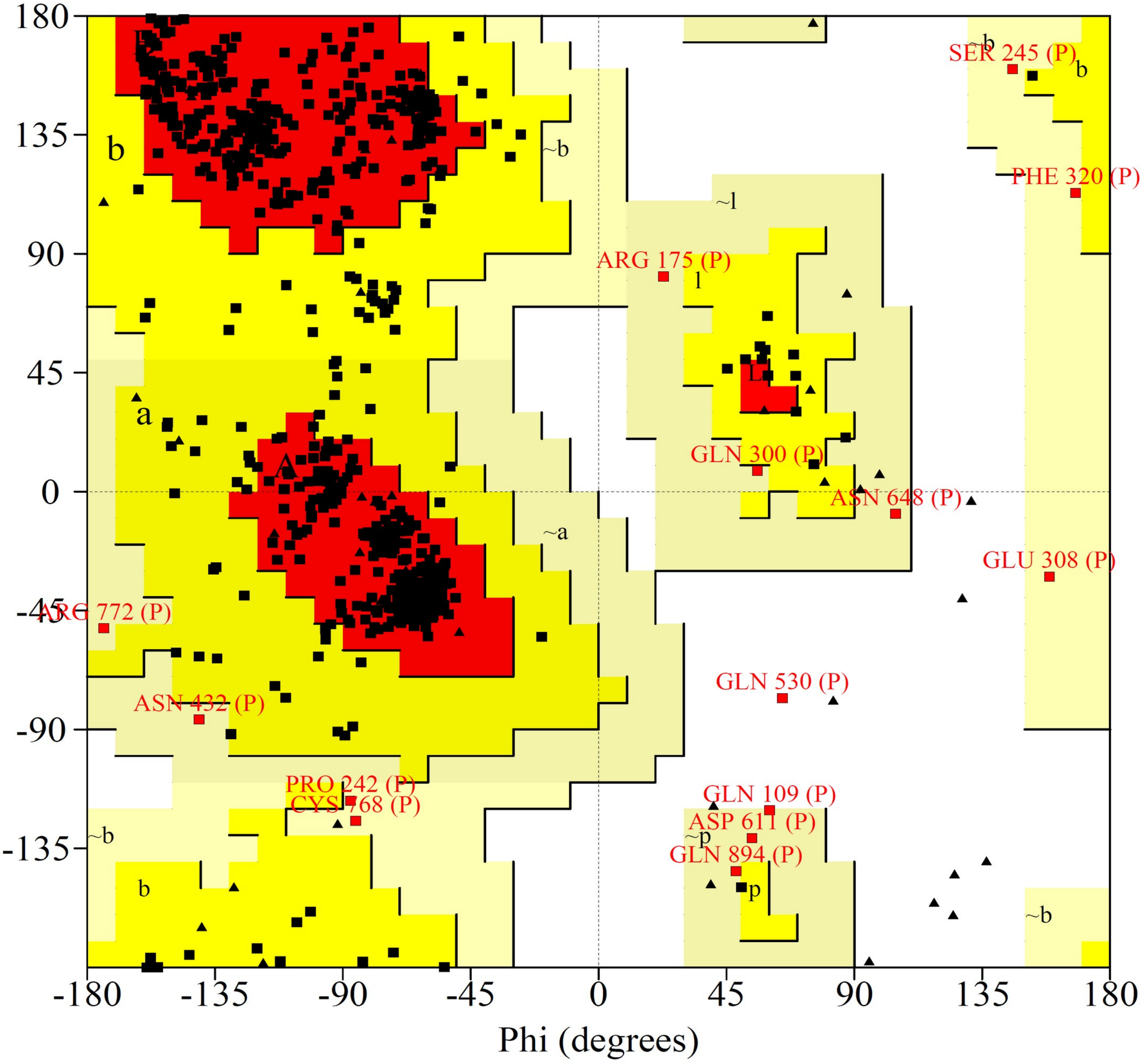

Plot statistics

Residues in most favoured regions [A,B,L]	705	86.5%
Residues in additional allowed regions [a,b,l,p]	97	11.9%
Residues in generously allowed regions [~a,~b,~l,~p]	12	1.5%
Residues in disallowed regions	1	0.1%
	----	------
Number of non-glycine and non-proline residues	815	100.0%
Number of end-residues (excl. Gly and Pro)	2	
Number of glycine residues (shown as triangles)	46	
Number of proline residues	32	

Total number of residues	895	
